# Where there are challenges, there are opportunities: An undergraduate medical students’ teaching concept for mental health in times of COVID-19

**DOI:** 10.1371/journal.pone.0277525

**Published:** 2022-11-10

**Authors:** Anne Herrmann-Werner, Rebecca Erschens, Stephan Zipfel, Teresa Festl-Wietek

**Affiliations:** 1 Medical Faculty Tuebingen, TIME (Tübingen Institute for Medical Education), Tuebingen, Germany; 2 Department of Internal Medicine VI/Psychosomatic Medicine and Psychotherapy, University Hospital Tuebingen, Tuebingen, Germany; University of Oxford, UNITED KINGDOM

## Abstract

COVID-19 had a tremendous effect on medical education. Most teaching sessions had to be shifted online, posing additional stress and potential isolation on medical students. However, it also offered the promotion of innovative digital teaching concepts. In this article, an approach to undergraduate mental health training is presented and evaluated. The curriculum was designed according to Kern’s six-step approach and consisted of asynchronous online material as well synchronous digital teaching and was accompanied by a plethora of newly developed teaching material (videos, fact sheets, etc.). Content covered the whole spectrum of diseases seen in a service of psychosomatic medicine and psychotherapy (i.e. anxiety, depression, trauma, somatoform and eating disorders, as well as motivational interviewing). Feedback from participants was collected, and exam results (written and practical) were compared to pre-COVID-19 times using t-tests for dependent and independent samples. Students were highly satisfied with the teaching (rating of 1.3 ± 0.6, n = 139 students). There was no significant difference from course evaluations before COVID-19 (1.5 ± 0.5, p > .05). The teaching also received an award in the students’ competition “best digital teaching concept in summer term 2020”. In the written exams, there was no significant difference between before COVID-19 (2.4 ± 0.45) and during COVID-19 times (1.6 ± 0.39; p > .05). In the practical objective structured clinical examination (OSCE), there was also no significant difference between students’ judgement of the difficulty of the station (1.9 ± 0.22 vs 1.9 ± 0.31; p > .05) or how well-prepared they felt for the exam (2.0 ± 0.24 vs 2.0 ± 0.31; p > .05). However, there was a significant difference in terms of grades, with the pre-COVID-19 grades being significantly better (2.7 ± 0.37 vs 2.0 ± 0.44; p < .05), which reflects the difficulty of transferring practical skills training to an online setting. Students particularly valued the possibility of self-directed learning combined with personal guidance by departmental experts, reflecting the importance of wellbeing-centred medical education. The pandemic triggered overnight challenges for teaching mental health that may also offer the opportunity to think about worldwide teaching standards with easily accessible material and courses online. This may offer the opportunity to enthral medical students to become mental health specialists themselves.

## Introduction

2020 started with what seems one of the biggest challenges for political, economic, medical and social contexts in modern history: The COVID-19 pandemic dramatically altered the world as we know it. People had to promptly adapt to ever-changing regulations and face unknown and difficult settings in all areas of life. This was also true for university teaching. In Germany, in mid-March 2020, the general lockdown was announced–only a month before the regular start of the academic summer term. It became clear that there was no chance for usual teaching, and faculties of medicine countrywide had to rapidly create solutions to adapt existing teaching formats to digital versions. Several national movements (e.g. https://hochschulforumdigitalisierung.de/, https://gesellschaft-medizinische-ausbildung.org/ausschuesse/digitalisierung-technologie-unterstuetztes-lernen-und-lehren/digitale-resourcen.html) tried to offer helpful emergency tool kits to bridge knowledge and competency gaps concerning digital tools. Internationally, the AMEE (Association of Medical Education in Europe) Technology Enhanced Learning committee published guidelines on how to swiftly migrate existing teaching into online versions [1].

Interestingly, this shift to pure digital teaching created a conflicting situation where on the one hand, medical students were put in the “safe environment” of impersonal online teaching, whilst on the other hand, they were sent prematurely into clinical settings to support frontline workers in the fight against COVID-19 [2–4]. The latter created excellent teaching opportunities and a sense of belonging but also fears and insecurities in medical students [5,6]. In order to keep the medical education running teaching courses were transferred into asynchronous or synchronous distance learning settings [7,8]. Additionally, the shift into digital teaching may increase feelings of loneliness and psychological impairment among students–particularly amongst medical students who are already prone to psychological stress and above-average rates of mental disorders [9,10]. Accordingly, faculties are asked to monitor for psychological needs or undesired course of events, especially in crisis situations and at least create teaching environments and material that are as supportive as possible without neglecting important content [11,12]. In its aims, the WPA Section on Education in Psychiatry defined standards for mental health teaching content and material [13]. It also acknowledged the importance of teaching for a general judgement on the subject, and more specifically, the influence of good teaching on medical students’ career choices [13]. The latter is particularly important as disciplines like psychosomatic medicine and psychiatry struggle to recruit junior staff to cover rising figures of mental health issues in the general population [14].

The Department of Psychosomatic Medicine and Psychotherapy, University Hospital Tuebingen, is part of the general Medical Department and has three areas of duty: 1) patient care, 2) research, and 3) teaching. The teaching responsibilities comprise a) subject-specific pathologies and corresponding psychotherapeutic interventions and b) overarching interpersonal skills (e.g. communication and interaction).

However, in times of Covid-19 communication skills were difficult to teach due to stringent governmental orders and the need to develop appropriate infection-control practices [15,16]. Moreover, mental health is a challenging topic to teach. Thus, the teaching course of Psychosomatic Medicine and Psychotherapy needed to be transferred into a digital interactive learning environment. Several authors suggested tips how to migrate online learning [1,17,18]. Synchronous distance learning was often the solution in order to teach students clinical or surgical technical skills [8,19]. But, is this solution also suitable when teaching mental health issues?

Previously, in Germany, online lectures or live broadcasts were implemented in the medical training when the pandemic started [20]. Innovative digital teaching strategies, including serious games and virtual-reality exercises, though, were missing [20].

### Aim

This article aims to show how our department reacted in transforming its teaching of mental health issues in times of COVID-19. It also shows adaptations that were made according to different stages of the pandemic. Besides an elaborate outline of the teaching structure and content, evaluation and exam results will be presented as outcome measures to show whether main competencies in psychosomatic medicine and psychotherapy can still be achieved under such adverse circumstances.

The following research questions arouse regarding this online teaching course:

- How can the teaching of mental health issues in times of Covid-19 take place?
- When rapidly migrating teaching into online settings, which adaptations are necessary when the class is run long-term?
- Does the new teaching concept affect the students’ exam performance including written exam and OSCE compared to face-to-face setting?

As hypotheses we assumed that the transfer of the teaching course is possible and that the teaching is accepted by the students, however, they will prefer the teaching in person. Finally, we hypothesize that the exam results will suffer.

## Methods

### Study design, setting and student population

This observational study investigated the discipline-specific course in psychosomatic medicine and psychotherapy (PMP) with the corresponding exams (written test with 20 MCQ and objective structured clinical examination [OSCE] station, taking a history from a patient presenting with depression or panic disorder or chronic pain or bulimia or irritable bowel syndrome, respectively) was taken by third-year medical students. Teaching was held in groups of 20 students each.

### Planning of teaching

According to Kern’s six-step approach to curriculum development, we conducted a needs assessment with relevant stakeholders, defined goals and objectives with aligned teaching strategies for the class and implemented the course immediately [21]. Several structures were established for evaluation and feedback: 1) oral feedback session in a synchronous meeting, 2) written feedback via the usual evaluation system of the faculty, and 3) frequent meetings with student representatives. Teaching was designed as a combination of guided gain of knowledge and competencies as well as self-directed learning.

### Evaluation and exams

Students evaluated the teaching class within the regular online evaluation tool (EvaSys, https://evasys.de/) after teaching had finished. At the OSCE, they also rated three specifically created items: 1) How appropriate they found the content and the level of difficulty for the station (*Content and Level of Difficulty*), 2) How well-prepared they felt by the PMP teaching for the OSCE exam in PMP (*Preparation*), and 3) Whether they felt fit to perform the content of the simulated OSCE station in real clinical life (*Fit to practice*). All ratings followed the German school grade system, with 1 = “very good” to 6 = “unsatisfactory”. Exam grades followed the same rating scheme and were given by expert examiners from the department of PMP. The questions were developed by using the think-aloud technique. They were self-reported and not validated. The performance in the written exam and in the OSCE was assessed in a standardised and validated format.

### Statistical analyses

Evaluation and exam results are presented as means and standard deviations. Normal distribution of the data was tested by using the Kolmogorov-Smirnov test. T-tests for independent and dependent samples were conducted to compare the results. The level of significance was p < .05. Statistical analyses were performed with SPSS for Windows version 25.0 under the assumption that the variables followed a normal distribution.

### Ethical approval

The survey received ethics approval from the Ethics Committee of Tuebingen Medical Faculty (no. 314/2020BO2).

## Results

### Teaching structure and content

#### Summer term 2020

Teaching started with an email invitation. It contained a welcome message, an informative outline on the whole class, including learning objectives and login details for the secured teaching content within the university’s learning management system (ILIAS, www.ilias.de). It also provided information about the specifically created online forum, which was checked for posts once a day by a teaching assistant who encouraged students to ask questions at any time.

Students were informed about the necessity to work through some online material asynchronously before their first synchronous teaching sessions. The material comprised recorded lectures on the major topics of the discipline (general information on the subject of PMP, motivational interviewing, eating disorders, anxiety disorders, depression, trauma-related disorders, somatic symptom disorders) with an accompanying online quiz to check the level of understanding, as well as fact sheets summarizing main points for all diseases covered. Additionally, in line with evidence-based medicine, national guidelines for all disorders taught were made available if existing. Students then met with their teacher online in groups of 20 using Zoom® professional. The first session was an interactive discussion on the theoretical knowledge regarding PMP and a preparation for the next asynchronous phase. This phase consisted of casework by the students. We had created ten paper-cases mirroring the most common mental health presentations in a German PMP department. Students had to pick two out of these ten cases, guided by leading questions about diagnosis, prognosis, treatment options and possible complications. They had to submit their cases dealt with, which were then corrected by experienced physicians from our department. Students received individual feedback on each case submitted.

Additionally, a commercial though due to COVID-19 free-of-charge software programme was used presenting a virtual hospital (INMEDEA, www.inmedea-simulator.net/med/scene/entry). Students chose one out of two predefined patient cases. In the gamified interaction, they took the patient’s history, conducted exams and tests and suggested a treatment plan. At the end of the case, the system offered feedback on the performance, including an explanation of incorrect answers and the total sum spent on the patient. The casework had to be done within a two-week window. At the end of this period, a second synchronous meeting took place. It was meant as an open discussion on the cases, sharing experience with the online learning situation and room for questions. It also presented the link to an ILIAS folder that contained exam preparation material, including learning sheets for the written exam and short video clips of physician-patient interactions for the OSCE covering all major topics and revealed the checklist as a basis for the grading. Students were encouraged to work through the material and offered an additional online meeting as specific practice for the OSCE. Within this final session, students could ask questions about the OSCE in general and subject-specific issues. They also had the opportunity to practice two exemplary stations with standardized patients (SP) and subsequent structured feedback, including displaying the underlying checklist to familiarize students with the expected performance and evaluation scale.

#### Adaptations in winter term 2020/21 and summer term 2021

Due to changes in local regulations and the introduction of rapid antigen testing possibilities, some mitigations of restrictions could take place. Still, only strictly necessary face-to-face teachings were allowed, so the PMP course continued to be entirely online. However, we started to mirror our pre-COVID-19 teaching and taught students synchronously for all six original sessions, re-introducing simulated talks with SP. The SP represented typical presentations of mental illnesses as examples for the respective topic of the day (bulimia, depression, panic disorder, PTSD, somatoform pain) or simulated a patient who had to be motivated for psychotherapy (motivational interviewing). Teaching followed the usual pattern of a theoretic input on the topic, the simulated session, and feedback from the group and the expert teacher. Besides PMP content, telemedical aspects of video-based physician-patient communication were discussed. Also, as the data protection possibilities of the video conferencing system (Zoom®) changed to end-to-end encryption, we could even introduce sessions with actual patients starting in the summer term 2021. This was implemented by a live-stream ward round where senior physicians of our department interviewed patients, followed by an interaction of students with the patients via real-time chat.

#### Course evaluation

In the oral feedback round, students expressed their appreciation of class format and content and were particularly grateful for the mixture of working independently while having easily accessible contact possibilities and a defined person in charge. They also highly valued the created material that helped them easily figure out relevant aspects of the subject. In the online evaluation, the course reached an average rating of 1.3 ± 0.6 (n = 139 students). There was no significant difference from course evaluations before COVID-19 (1.5 ± 0.5, p > .05). The teaching also received an award in the students’ competition “best digital teaching concept in summer term 2020”.

#### Written exam

In the summer term 2020, n = 167 students took the written exam. The average grade was 1.31 (SD 0.64), and only one student failed the exam. In winter term 2020/21, n = 168 students took the written exam. The average grade was 1.86 (SD 0.91). Again, only one student failed the exam. There was no significant difference between the two written exams before COVID-19 (2.4 ± 0.45) and the two in COVID-19 times (1.6 ± 0.39; p > .05).

#### Objective structured clinical examination

In the summer term 2020, n = 149 students took the OSCE as a face-to-face exam under strict hygienic measurements. A more detailed description of the entire face-to-face OSCE in the pandemic will be published elsewhere (currently under review in Medical Teacher).

Students evaluated the OSCE in all three dimensions as good and with results comparable to before COVID-19 (Table 1).

**Table 1:** Results of OSCE evaluation by students following the German school grade system with 1 = “very good” to 6 = “unsatisfactory”. Values of 1) content and level of difficulty, 2) feeling prepared for the OSCE station and 3) ability to perform the task in reality are shown as Mean and SD for the subject of Psychosomatic Medicine and Psychotherapy in the COVID-19 OSCE (10/2020). Comparison for 1) and 2) are shown regarding pre-COVID-19 OSCE. Comparison for item 3) was not possible as it had not been asked before the pandemic.

	OSCE PMP in COVID-19	OSCE PMP before COVID-19	Statistics
1) Content and Level of difficulty	1.9 ± 0.22	1.9 ± 0.31	p > .05
2) Preparation	2.0 ± 0.24	2.0 ± 0.31	p > .05
3) Fit to practice	2.1 ± 0.35	n.a.	n.a.

However, there was a statistically significant difference in grades given by the PMP examiners in the OSCE exam in times of COVID-19 as opposed to the OSCE exams before (Table 2).

**Table 2:** Results of OSCE evaluation by experts of Psychosomatic Medicine and Psychotherapy following the German school grade system with 1 = “very good” to 6 = “unsatisfactory”.

	OSCE PMP in COVID-19	OSCE PMP before COVID-19	Statistics
Grade in PMP station	2.7 ± 0.37	2.0 ± 0.44	p < .05

The OSCEs for students from winter term 20/21 and summer term 21 are pending.

## Discussion

COVID-19 was a big challenge for many areas. In teaching, subjects with an intense patient-physician relationship had to find ways to transfer their courses to online versions. Regarding the research questions our PMP department rapidly created an online version of its teaching in summer term 2020 following guidelines and curriculum design instruments [1,21]. An interactive online learning setting was used to teach mental health issues and communication skills. Furthermore, telemedical aspects of video-based physician-patient communication were discussed. The course was highly appreciated by students, particularly because of the human interaction, even in the complete online version of the class, and because of the easy-to-understand created material reflected main facts on mental health.

In contrast to our hypotheses, exam results showed that the knowledge transfer was feasible without loss of performance compared to pre-pandemic written exams. In the practical exam (OSCE), this also accounted for the students’ view regarding difficulty. They felt equally well-prepared; however, the actual grades given by examiners from the PMP department were significantly worse than before the pandemic. This might indicate that particularly practical skills as tested in an OSCE may not be as easily transferable as factual knowledge. This is in line with the general acknowledgment that manual skills cannot be taught completely online [1]. On the other hand, the pandemic also offered the opportunity to start preparing students for telemedical consultations, helping them to naturally incorporate communicative and interactional skills required in telemedicine [22]. Through lively interactive discussions, students in our programme engaged with this virtual way of patient care. However, innovative digital teaching strategies like serious games or virtual-reality exercises are difficult to implement and need more time [20].

The true effects of the pandemic on medical students’ mental wellbeing remain to be determined [23]. In our study, students were grateful for the mixture of self-directed learning and synchronous guidance by experienced departmental staff. Coaching and an educational focus on the relationship with each student seems to be a key component in wellbeing-centred medical education [12,24].

Given the specifically created new online material, there is also the new opportunity to share learning resources worldwide and distribute gold-standard material even into the remotest areas, as suggested by central institutions [13,25]. This may be the right moment to define easily accessible worldwide teaching standards for undergraduate medical education in mental health. Teaching offers a good opportunity for transparency regarding what PMP does as well as to enthral medical students to choose a career in mental health [13]. This will be particularly important, as besides the already known shortage of mental health doctors compared to rising figures of patients with mental health issues, COVID-19 produced a significant rise in mental health problems worldwide [19].

## Limitations

Although we are aware that the programme described in this study was at one medical faculty only and with altering teaching conditions due to changes in the pandemic, we firmly believe that this observational study makes a valuable contribution to conceptualising mental health teaching formats for undergraduate medical students in a pandemic and beyond. However, we used self-developed questions in order to evaluate the teaching concept and not validated instruments.

## Conclusion

This study is the first to show a successful transfer of mental health teaching in response to COVID-19 challenges into an online format, including self-directed learning elements and preservation of social contact with teachers. Currently, we are performing a thorough scrutiny to decide which of the rapidly migrated online teaching components is worth keeping, even if there is an eventual return to teaching as before COVID-19. Further research should look at the success of such hybrid teaching options in the context of mental health to continue with the best of the two worlds.

## Abbreviations

AMEE: Association of Medical Education in Europe
PMP: Psychosomatic Medicine and Psychotherapy
SP: Standardized Patient
